# A case report: pitfalls in antibacterial therapy with rifampicin for mechanical valve endocarditis—the king of drug interactions

**DOI:** 10.1093/ehjcr/ytae525

**Published:** 2024-09-23

**Authors:** Ryosuke Honda, Yusuke Akazawa, Katsuji Inoue, Takashi Higaki, Osamu Yamaguchi

**Affiliations:** Department of Cardiology, Pulmonology, Hypertension, and Nephrology, Ehime University Graduate School of Medicine, Toon, Ehime, 791-0295, Japan; Department of Cardiology, Pulmonology, Hypertension, and Nephrology, Ehime University Graduate School of Medicine, Toon, Ehime, 791-0295, Japan; Department of Cardiology, Pulmonology, Hypertension, and Nephrology, Ehime University Graduate School of Medicine, Toon, Ehime, 791-0295, Japan; Department of Community Emergency Medicine, Ehime University Graduate School of Medicine, Yawatahama, Ehime, 796-8502, Japan; Department of Regional Pediatrics and Perinatology, Ehime University Graduate School of Medicine, Toon, Ehime, 791-0295, Japan; Department of Cardiology, Pulmonology, Hypertension, and Nephrology, Ehime University Graduate School of Medicine, Toon, Ehime, 791-0295, Japan

**Keywords:** Mechanical valve endocarditis, Rifampicin, Drug–drug interactions, Tolvaptan, Case report

## Abstract

**Background:**

Rifampicin is a strong inducer of the hepatic cytochrome P450 (CYP) family and is known to interact with many clinical drugs. However, to our knowledge, no case of worsening heart failure (HF) due to the interaction between rifampicin and HF drugs has been reported.

**Case summary:**

A 32-year-old female, who had undergone intracardiac repair for an incomplete atrioventricular septal defect with dextrocardia and prosthetic valve replacements for right and left atrioventricular valve regurgitation, presented as an outpatient. Her medications included tolvaptan 15 mg and warfarin 1.25 mg. She had a slight fever and Osler nodes at her fingers. Blood culture bottles grew methicillin-resistant *Staphylococcus epidermidis*, and several vegetations were observed on the right atrioventricular mechanical valve with a transoesophageal echocardiogram. She was diagnosed with prosthetic valve endocarditis and treated with antibiotic agents including rifampicin. After a week, she developed systemic oedema and had a marked decrease in prothrombin time–international normalized ratio (PT-INR). Rifampicin was promptly discontinued due to a strong suspicion of a drug–drug interaction. Consequently, both her congestion and the PT-INR stabilized, and she was discharged after 8 weeks of antibiotic treatment.

**Discussion:**

The introduction of rifampicin induces CYP family members such as CYP3A4 and CYP2C9. Warfarin is metabolized by CYP2C9 and tolvaptan is also metabolized by CYP3A4, resulting in a notable reduction of their blood levels when co-administered with rifampicin. The clinical challenges arising from interactions between HF drugs and rifampicin can be categorized into two main groups: worsening HF and thrombotic complications. Clinicians should remain vigilant and informed about these potential issues.

**Learning points**

- The novelty of this paper lies in the lack of clinical reports on the interaction of tolvaptan and rifampicin, and in the demonstration of the ineffectiveness of switching to intravenous tolvaptan.
- Clinicians should be aware of the worsening HF and resistance to anticoagulation therapy due to drug–drug interactions of rifampicin in patients with prosthetic valve infectious endocarditis.

## Introduction

Adult patients with congenital heart disease (ACHD) frequently develop heart failure (HF) at a young age and require polypharmacy as part of their life-long management. Moreover, these patients have an elevated risk of developing infective endocarditis (IE), further increasing the potential for adverse drug–drug interactions.

Rifampicin is known to strongly induce the expression of the cytochrome P450 (CYP) family, including CYP3A4, which leads to interactions with many cardiovascular drugs. In this case report, we highlight a patient with ACHD and chronic HF who underwent mechanical valve replacement and developed worsening HF as well as resistance to anticoagulation therapy due to a significant drug–drug interaction between tolvaptan and warfarin after starting rifampicin to treat IE.

## Summary figure

**Figure:**
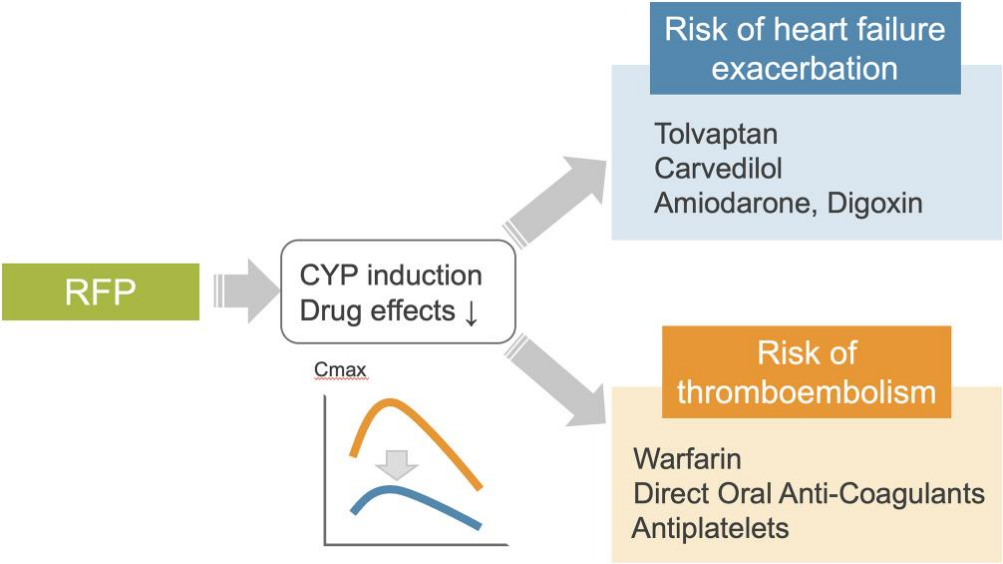


## Case presentation

**Figure 1:**
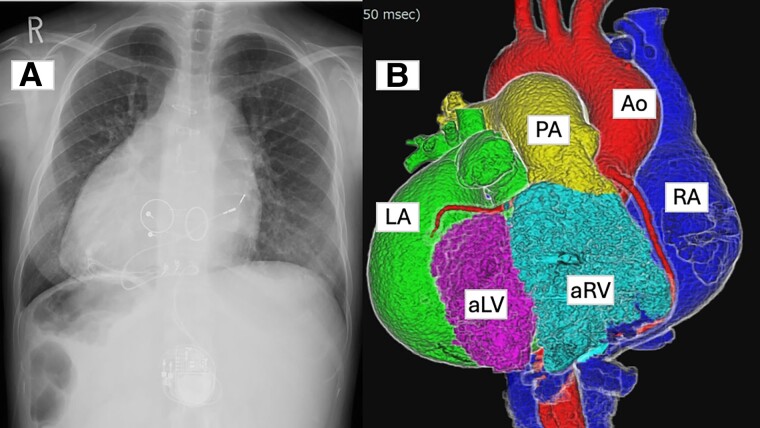
Chest radiography (*A*) and cardiac computed tomography (*B*) showing dextrocardia and incomplete atrioventricular septal defect. Ao, aorta; aLV, anatomical left ventricular; aRV, anatomical right ventricular; LA, left atrium; PA, pulmonary artery; RA, right atrium.

**Figure 2:**
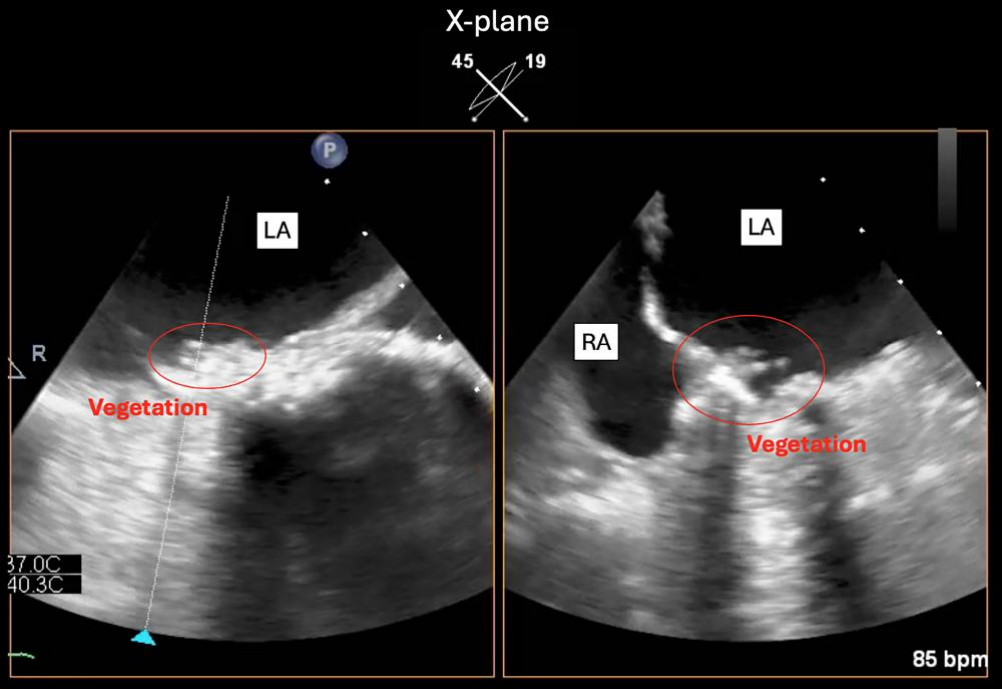
Transoesophageal echocardiogram (*x*-plane mode with 45° and 19° angles) showing vegetations on the right atrioventricular mechanical valve (in the circle). LA, left atrium; RA, right atrium.

**Figure 3:**
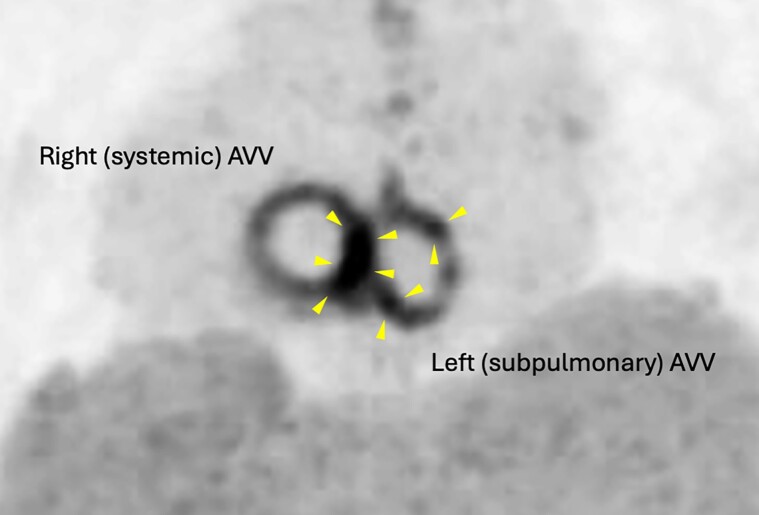
^18^F-Fuorodeoxyglucose positron emission tomography/computed tomography showing ^18^F-ﬂuorodeoxyglucose uptake with nodules (arrowheads). AVV, atrioventricular valve.

A 32-year-old woman with a complex medical history, including intracardiac repair for dextrocardia, incomplete atrioventricular septal defect, and partial anomalous pulmonary venous return, presented to the outpatient department for routine medical examination. We performed mechanical valve replacement for severe right (systemic) and left (subpulmonary) atrioventricular valve regurgitation, as well as epicardial pacemaker implantation for complete atrioventricular block (*Figure 1*). She had taken warfarin 1.25 mg, tolvaptan 15 mg, furosemide 80 mg, dapagliflozin 10 mg, sacubitril/valsartan 24.3/25.7 mg, bisoprolol 1.25 mg, and eplerenone 50 mg, as prescribed. She had painful spots on her finger ends with a slight fever and had Osler’s nodes on her bilateral finger ends. Two sets of blood cultures showed the growth of methicillin-resistant *Staphylococcus epidermidis*, which was also resistant to gentamicin (GM). Laboratory testing showed the following results: albumin of 3.4 g/dL, total bilirubin of 0.9 mg/dL, C-reactive protein of 0.37 mg/dL, and a prothrombin time–international normalized ratio (PT-INR) of 2.01. Transthoracic echocardiography showed a preserved ejection fraction in the systemic ventricle, but estimation of diastolic capacity was challenging due to atrial fibrillation. No evidence of prosthetic valve detachment, stuck valve, or exacerbated regurgitation was observed. The mean pressure gradients of the anatomical left and right atrioventricular mechanical valves were not elevated (4.8 and 4.3 mmHg). Transoesophageal echocardiography showed that the right atrioventricular mechanical valve had several mobile vegetations that were 4 mm in size on the suture ring (*Figure 2*, Supplementary material online, *Video S1*). ^18^F-Fuorodeoxyglucose positron emission tomography/computed tomography (^18^F-FDG PET/CT) showed ^18^F-FDG uptake by nodules on the right and left prosthetic valve annuluses, with the maximum standardized uptake value (SUV_max_) of 8.24 (*Figure 3*). Contrast-enhanced CT showed no evidence of septic emboli. Therefore, she was diagnosed with prosthetic valve endocarditis (PVE). Daily administration of vancomycin 2000 mg instead of surgical intervention was initiated given the absence of significant circulatory compromise, including valve dysfunction and the small mass.

However, with her fever and re-elevation of the inflammatory response, we found the infection difficult to control, so based on European Society of Cardiology guidelines,^1^ rifampicin 600 mg was added after 3 days. One week later, her weight had gradually increased from 42 to 50.7 kg, accompanied by an elevation in total bilirubin to 3.1 mg/dL, indicative of systemic congestion. Additionally, her PT-INR had markedly decreased from 2.37 to 1.43.

Transthoracic echocardiography revealed no significant alterations, with the mean pressure gradients across the anatomical left and right atrioventricular valves remaining stable at 5.5 and 4.5 mmHg with mild regurgitation, respectively. Right heart catheterization was not feasible because the atrioventricular valve was mechanical and the catheter could not be inserted beyond the right atrium. The central venous pressure caused by a peripherally inserted central venous catheter was 15 mmHg.

**Figure 4:**
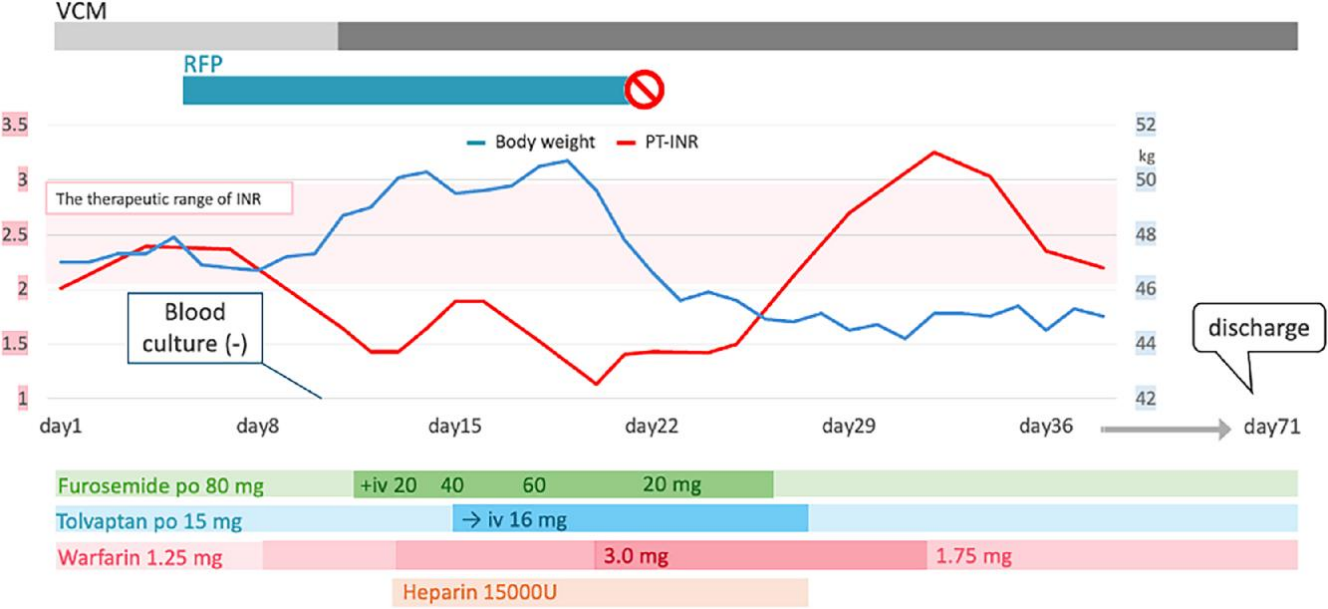
Daily heart failure drug dosage, international normalized ratio values, body weight, and antibacterial therapy over time. The *x*-axis represents the date of admission. The left *y*-axis represents the prothrombin time–international normalized ratio values (therapeutic range: 2.0–3.0). The right *y*-axis represents the body weight, shown by the blue line. The time period of the antibacterial therapy is indicated by the horizontal bar at the top of the graph. The type, dosage, and duration of heart failure medications are indicated by the horizontal bars at the bottom of the graph. RFP, rifampicin; VCM, vancomycin.

Switching oral tolvaptan and loop diuretics to intravenous administration along with increasing the warfarin dosage was ineffective. During the ineffective warfarin therapy period, heparin as an anticoagulant therapy was administered concomitantly. Suspecting a drug–drug interaction, we discontinued rifampicin. Subsequently, her PT-INR was prolonged to an appropriate degree, and her fluid balance was maintained with oral tolvaptan and furosemide. After treatment with vancomycin for 8 weeks, transoesophageal echocardiography showed regression of vegetation and decrease in the SUV_max_ to 6.7 on ^18^F-FDG PET/CT. She was discharged, and no deterioration was observed in the outpatient 6-month follow-up (*Figure 4*).

## Discussion

Rifampicin interacts with numerous drugs, which requires cautious administration by clinicians. We treated a patient with ACHD who was on multiple HF medications and antithrombotic agents following cardiac surgery, which included mechanical valve replacement. Notably, her HF worsened due to an interaction with tolvaptan ∼1 week after initiating rifampicin. Clinical examinations ruled out the possibility that vegetation-induced valve dysfunction or other factors contributed to the decompensation of HF.

Rifampicin is completely absorbed and widely distributed in most bodily tissues and fluids^2^ and exerts a bacteriostatic effect through DNA-dependent RNA polymerase inhibition. Guidelines for IE^1^ have recommended combining rifampicin and GM with anti-methicillin-resistant *S. aureus* drugs as a treatment option for PVE owing to *Staphylococcus* species. One advantage of rifampicin for PVE is its ability to penetrate the biofilm that *Staphylococcus* forms on artefact surfaces,^3^ as determined from *in vitro* and *in vivo*^4,5^ investigations.

Drug interactions are a clinical pitfall in rifampicin treatment and occur through the ligand-dependent transcription factor pregnane X receptor that is activated by rifampicin binding.^6^ Rae *et al*. reported an increase in CYP2C9 and CYP3A4 RNA in cells with added rifampicin. Rifampicin increased RNA expression of CYP2C9 3.7-fold and, surprisingly, increased CYP3A4 up to 55.1-fold,^7^ which is involved in the metabolism of 50%–60% of all clinical drugs.

It is widely recognized that warfarin undergoes predominant metabolism through CYP2C9, whereas tolvaptan is metabolized by CYP3A4.^8^ Reportedly, the blood concentration of tolvaptan decreased to only 13% during rifampicin treatment.^9^ In patients with HF, decreased drug absorption due to gastrointestinal oedema may affect bioavailability. Although intravenous tolvaptan is available, it was ineffective in this case, leading us to suspect a drug–drug interaction.

Most patients with ACHD who develop IE have undergone open heart surgery and have prosthetic valves or other prosthetic replacements. These patients are already experiencing chronic HF and are taking numerous medications, including cardioprotective agents, antithrombotic agents, and diuretics. Tolvaptan, digoxin, carvedilol, amiodarone, and apixaban are metabolized by CYP3A4, and warfarin is metabolized by CYP2C9. Full induction of drug-metabolizing enzymes is reached in ∼1 week after starting rifampicin treatment, and the induction dissipates within 2 weeks after discontinuation.^10^ Interactions with rifampicin reduce the efficacy of these drugs and manifest clinically as (i) worsening HF and (ii) thrombotic complications. Clinicians should always be alert to such interactions and may need to choose rifampicin-free regimens.

## Lead author biography


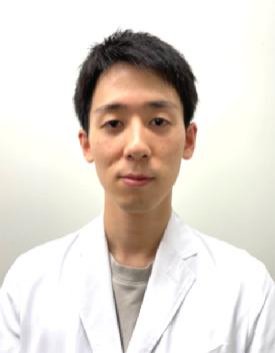


Dr Ryosuke Honda is working as a cardiologist in the Department of Cardiology, Pulmonology, Hypertension, and Nephrology, Ehime University Graduate School of Medicine, in Japan. He specializes in internal medicine and cardiovascular disease.

## Supplementary Material

ytae525_Supplementary_Data

## Data Availability

The data supporting this article are available in the article and in its Supplementary material online.

## Notes

**Consent:** The authors confirm that written consent for submission and publication of this case report including images and associated text has been obtained from the patient in line with COPE guidance.

**Funding:** None declared.
